# PlantPAN 4.0: updated database for identifying conserved non-coding sequences and exploring dynamic transcriptional regulation in plant promoters

**DOI:** 10.1093/nar/gkad945

**Published:** 2023-10-28

**Authors:** Chi-Nga Chow, Chien-Wen Yang, Nai-Yun Wu, Hung-Teng Wang, Kuan-Chieh Tseng, Yu-Hsuan Chiu, Tzong-Yi Lee, Wen-Chi Chang

**Affiliations:** Institute of Tropical Plant Sciences and Microbiology, College of Biosciences and Biotechnology, National Cheng Kung University, Tainan 701, Taiwan; School of Molecular Sciences, Arizona State University, Tempe 85281, USA; Institute of Tropical Plant Sciences and Microbiology, College of Biosciences and Biotechnology, National Cheng Kung University, Tainan 701, Taiwan; Institute of Tropical Plant Sciences and Microbiology, College of Biosciences and Biotechnology, National Cheng Kung University, Tainan 701, Taiwan; Institute of Tropical Plant Sciences and Microbiology, College of Biosciences and Biotechnology, National Cheng Kung University, Tainan 701, Taiwan; Department of Life Sciences, National Cheng Kung University, Tainan 701, Taiwan; Graduate Program in Translational Agricultural Sciences, National Cheng Kung University and Academia Sinica, Tainan 701, Taiwan; Department of Biological Science & Technology, National Yang Ming Chiao Tung University, Hsinchu 300, Taiwan; Institute of Tropical Plant Sciences and Microbiology, College of Biosciences and Biotechnology, National Cheng Kung University, Tainan 701, Taiwan; Department of Life Sciences, National Cheng Kung University, Tainan 701, Taiwan; Graduate Program in Translational Agricultural Sciences, National Cheng Kung University and Academia Sinica, Tainan 701, Taiwan

## Abstract

PlantPAN 4.0 (http://PlantPAN.itps.ncku.edu.tw/) is an integrative resource for constructing transcriptional regulatory networks for diverse plant species. In this release, the gene annotation and promoter sequences were expanded to cover 115 species. PlantPAN 4.0 can help users characterize the evolutionary differences and similarities among *cis-*regulatory elements; furthermore, this system can now help in identification of conserved non-coding sequences among homologous genes. The updated transcription factor binding site repository contains 3428 nonredundant matrices for 18305 transcription factors; this expansion helps in exploration of combinational and nucleotide variants of *cis-*regulatory elements in conserved non-coding sequences. Additionally, the genomic landscapes of regulatory factors were manually updated, and ChIP-seq data sets derived from a single-cell green alga (*Chlamydomonas reinhardtii*) were added. Furthermore, the statistical review and graphical analysis components were improved to offer intelligible information through ChIP-seq data analysis. These improvements included easy-to-read experimental condition clusters, searchable gene-centered interfaces for the identification of promoter regions’ binding preferences by considering experimental condition clusters and peak visualization for all regulatory factors, and the 20 most significantly enriched gene ontology functions for regulatory factors. Thus, PlantPAN 4.0 can effectively reconstruct gene regulatory networks and help compare genomic *cis-*regulatory elements across plant species and experiments.

## Graphical Abstract

**Graphical Abstract:**
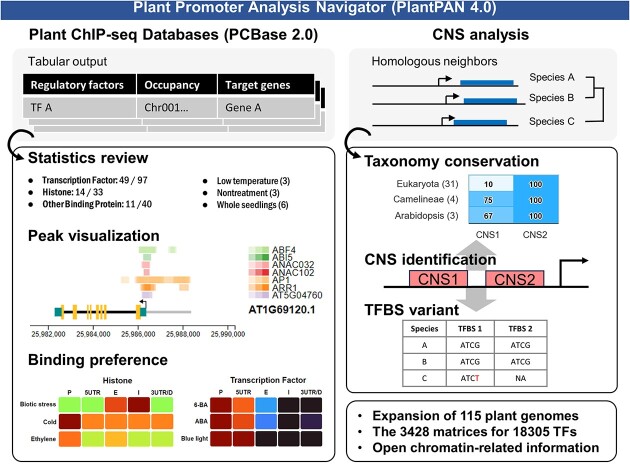

## Introduction

The transcription of protein-coding and non-coding genes is regulated by multiple regulatory factors (RFs), which may work coordinately or antagonistically. Transcription factors (TFs), which constitute a major group of key transcription regulators, typically recognize and bind to specific sequences located upstream of their target genes. However, a computational and conceptual challenge in accurately predicting TF binding sites (TFBSs) is that the known TFBSs are short sequences (4–12 bp) and exhibit tolerance to nucleotide variants. These properties result in a high rate of false-positive results and numerous candidates during the prediction of TFBSs. Conserved non-coding sequences (CNSs) may highlight putative *cis-*regulatory elements in the non-coding regions of genomes (1). Enhancers or experimentally verified TFBSs, which have been identified within animal CNSs, play key regulatory roles during developmental stages and in tissue-specific transcription (2–4). Plant CNSs have been used as indicators of shared regulations and evolutionary divergence between homologous genes of two species or within specific lineages, such as *Brassicaceae* and *Solanaceae* (5,6). This implies that CNSs exhibit consistent sequence characteristics within plant lineages, which facilitates the effective transfer (and validation) of gene regulation patterns observed in a model organism to another model organism or a non-model organism. For the systematic analysis of CNSs, CNS-related databases have been developed to identify CNSs across animals and plants (7,8). However, the current CNS-related databases do not facilitate the identification of *cis-*regulatory elements or the prediction of TFBSs in CNSs.

The extensive collection of ChIP-seq data may help in the exploration of complex gene regulatory networks through comparative analysis of genomic occupancy. ChIP-seq peak clusters may shed light on co-regulation or competition between RFs (9,10). The occupancy landscapes of RFs change during different development stages, within various tissues and in response to environmental stimuli, and RFs play diverse roles in mediating several biological processes (11,12). To help users explore the coordinated regulation between various regulators and the dynamic regulation across various conditions, several ChIP-seq-related databases offer integrated data analysis and graphical data visualization tools, which enable users to compare genomic occupancy across multiple ChIP-seq data sets (13,14). However, these tools were developed with consideration of only the animal kingdom. To the best of our knowledge, no resource is available for conducting a systemic comparison and analysis of plant ChIP-seq data sets.

Herein, we describe the updated version of the Plant Promoter Analysis Navigator system (PlantPAN 4.0), which can fill the gap pertaining to the systematic analysis of gene regulation in plants. The updated platform has a new function, CNS analysis; thus, it can now facilitate identification of CNSs among homologous genes across 115 plant species. The CNS analysis currently incorporates plant phylogenetic trees and plant taxonomy to elucidate evolutionary conservation among homologous genes and CNSs. Furthermore, the updated TFBS data, which cover 3428 nonredundant matrices for 18305 TFs, allow users to explore the regulation of TFs and the nucleotide variants of TFBSs across homologous genes. To improve the utility and explainability of ChIP-seq data, a tagging system was incorporated into PlantPAN 4.0 for the classification of disorganized ChIP-seq sample descriptions into easy-to-read clusters. These clusters can be used to analyze the 20 significantly enriched gene ontology (GO) functions for RFs (e.g. TFs, histones and other DNA-binding proteins) and to define the binding preferences of promoter regions for genes. The statistical review and graphical analysis components of the database help users effectively explore the dynamic regulation of a given RF or gene. PlantPAN 4.0 also help researchers identify potential complex regulatory networks involving multiple RFs and various experimental conditions.

## Data content and web interface

### Expansion of data on plant genomes and TF–TFBS matrices

The updated PlantPAN 4.0 databases offers extensive data on plant genomes; the number of plant species covered by this database has been increased from 7 to 115 (Supplementary Table S1). For the 4.0 update, the genome sequence and gene annotation data of *Arabidopsis thaliana*, *Oryza sativa* and *Zea mays* were obtained from the following resources, respectively: The *Arabidopsis* Information Resource, Rice Annotation Project Database and Maize Genetics and Genomics Database (15–17). Corresponding data for the other 112 plant species were downloaded from Ensembl Plants (release 52) and Phytozome (version 13) (18,19). The versions of plant genomes and the numbers of protein-coding and non-coding genes used in PlantPAN 4.0 are presented in Supplementary Table S1. To expand the data on genomic regulatory states, information on the genomic landscapes of open chromatin regions (derived from ChIP-hub and PlantRegMap) was incorporated into PlantPAN 4.0 (8,20); for this, only the genome versions that matched those present in PlantPAN 4.0 were used. Open chromatin-related information is currently available for 11 plant species. The revised database includes updated data on TFBS matrices, which were obtained from the following two resources: Just Another Splice Alignment Server and Catalog of Inferred Sequence Binding Preferences (version 2.00) (21,22). In PlantPAN 4.0, the total numbers of TFs and their corresponding matrices are 18 305 and 3428, respectively.

### Identification of homologous neighbors and CNSs

PlantPAN 4.0 has a new function that enables users to identify the CNSs located upstream or downstream of a gene of interest. Given that public resources contain homologous gene-related information for only a limited number of plant species, homologous neighbors were identified among 115 plant species by using InParanoid-DIAMOND with the default cutoff parameter setting (23). Technically, InParanoid-DIAMOND defined the homologous genes of a pair of plant species on the basis of protein (amino acid) sequences similarity. The homologous gene group for a given plant species comprised all homologous neighbor genes from the other 114 species and genes from its own species that shared the same homologous neighbors. The numbers and percentages of protein-coding genes with homologous neighbors are listed in Supplementary Table S1. To reveal the functional properties that are common among homologous genes, the InterProScan tool was used to identify protein domains, repeats, families and motifs (24). The MUSCLE 3.7 tool and the ‘ape 5.7’ R package were used to draw a phylogenetic tree (25,26).

To identify CNSs for a given gene, its promoter sequence—regions located upstream and downstream of the gene—were aligned against those of its homologous neighbors by using the Basic Local Alignment Search Tool (27). The following four user-defined parameters were included for CNS identification: sequence identity, e-value (Basic Local Alignment Search Tool) cutoff, minimum CNS length and the minimum number of species that could be aligned to the target CNSs. To determine the extent to which evolutional lineages shared the identified CNSs, the taxonomic lineages of 115 plant species were obtained from the Taxonomy database (National Center for Biotechnology Information) (28). Evolutionary conservation of CNSs within each plant lineage was visualized using Highcharts (https://www.highcharts.com/). The TFBSs in each CNS were predicted using the TFBS prediction tool that was incorporated into PlantPAN3.0 (29).

### Update of ChIP-seq data and experimental condition clusters

All experimental ChIP-seq data present in PCBase 2.0 (a subdatabase of PlantPAN 4.0) were retrieved from the Gene Expression Omnibus and Sequence Read Archive (30,31); in this retrieval, the following criteria were applied: (i) ChIP-seq experimental sample with its corresponding input (control), (ii) availability of raw data in the Sequence Read Archive or FASTQ format, (iii) a complete and clear description of experimental conditions and methods and (iv) wild types or rescued lines. All data were manually curated and organized into a unified format. The processing of ChIP-seq data and the *de novo* motif discovery of TF samples were optimized on the basis of relevant study (Supplementary Data) (29). A total of 580 data sets (1982 samples) were systemically analyzed to identify transcriptional relationships between 205 RFs across the following eight plant species: *A. thaliana, A. lyrata*, *O. sativa*, *Glycine max*, *Solanum lycopersicum*, *Gossypium hirsutum*, *Z. mays* and *Chlamydomonas reinhardtii*. To enhance the utility and interpretability of the results of ChIP-seq data analysis, a tagging system was incorporated into PCBase 2.0 for the classification of disorganized ChIP-seq sample descriptions into easy-to-read clusters, which were used for subsequent functional enrichment analysis and binding preference exploration. For instance, a data set comprising the results of a 16°C treatment and that comprising the results of a 17°C treatment were classified into the same treatment cluster, which bore the tag ‘low temperature’.

### Functional enrichment analysis in Protein Search function

To help users explore the biological functions of RFs, the GO term enrichment analysis function was incorporated into the Protein Search mode of PCBase 2.0. The GO annotations of the 115 species were downloaded using the BioMart tool within the Ensembl Plants, Phytozome database, or other public databases (18,19,32). The target genes identified through the analysis of ChIP-seq peak data were classified in the accordance with treatment tags. GO enrichment was evaluated using the hypergeometric distribution method (33). The dhyper () and phyper () functions in R were used to obtain hypergeometric *P* values for each set of target genes. Regarding *P* values, the cutoff was set at 0.05. All target genes identified from ChIP-seq data sets related to a given RF were incorporated into ‘all’ treatment. Only the 20 most enriched GO terms in ‘all’ treatment were visualized using a heat map.

## Application and discussion

### Overview of the improvements to PlantPAN

With the rapid expansion of genome sequence and gene annotation data available in public databases, conducting *cis-* and *trans-*regulatory element assays to study promoter regulation in plant species has become essential. PlantPAN 4.0 is effective tool in this context. The aforementioned database offers the genomic information of 115 plant species and has various integrated functions—Gene Search, Gene Group and Cross Species—for the analysis of these species. These functions can facilitate the identification of *cis-*regulatory elements (i.e. TFBSs, CpG islands and tandem repeats) within promoter regions, assessment of TF cooccurrence within a group of genes and analysis of the evolutionary conservation of CNSs and TFBSs. To help users identify dynamic regulatory regions before they conduct a promoter assay, the genomic landscapes of open chromatin regions derived from 769 publicly available samples of 11 plant species were incorporated into the basic gene information.

On the basis of information pertaining to the sequences of plant genomes and the key regulatory roles of CNSs, a new function—CNS analysis—was included within Cross Species function of the database to facilitate the identification of CNSs within promoter regions and offer insights into TF-based regulation across plant species. Another major improvement in PlantPAN 4.0 relates to the redesign of the framework for ChIP-seq data integration. Unlike the current ChIP-seq-related databases, which offer only one data set or have a tabular output format, PCBase 2.0 (the Plant ChIP-seq Database) facilitates the statistical overview of an input regulator or gene to help users rapidly understand the data that have been collected and the available information (29). Additionally, graphical results pertaining to peak visualization and binding preferences highlight regulatory differences in RFs and experimental conditions. To better illustrate the applicability of CNS analysis and PCBase 2.0, we present the following three case studies: evolutionary conservation of aluminum-activated malate transporter 1 gene (*ALMT1*, AT1G08430.1) regulation, gene-centered information on *APETALA1* (*AP1*, AT1G69120) and functional enrichment analysis of FLOWERING LOCUS M (FLM, AT1G77080) regulation in *A. thaliana*.

### Interfaces and applications of CNS analysis

CNS analysis can help identify highly conserved regions within the promoters of plant evolutionary lineages and help construct their potential TF-based regulations. Herein, we present a case study of a member of the ALMT family to clarify the visualization and application of CNS analysis (34). ALMT1 plays a role in malate efflux from roots and in the prevention of root growth inhibition due to the presence of toxic aluminum cations in acidic soils (34). Thus, ALMT1 is associated with an increase in crops’ aluminum or acid tolerance (35–38). However, TF-based regulations of *ALMT1* expression have been reported in a few plant species (39,40); this implies that the evolutionary conservation of gene regulation of ALMT1 remains rarely unknown.

**Figure 1.**
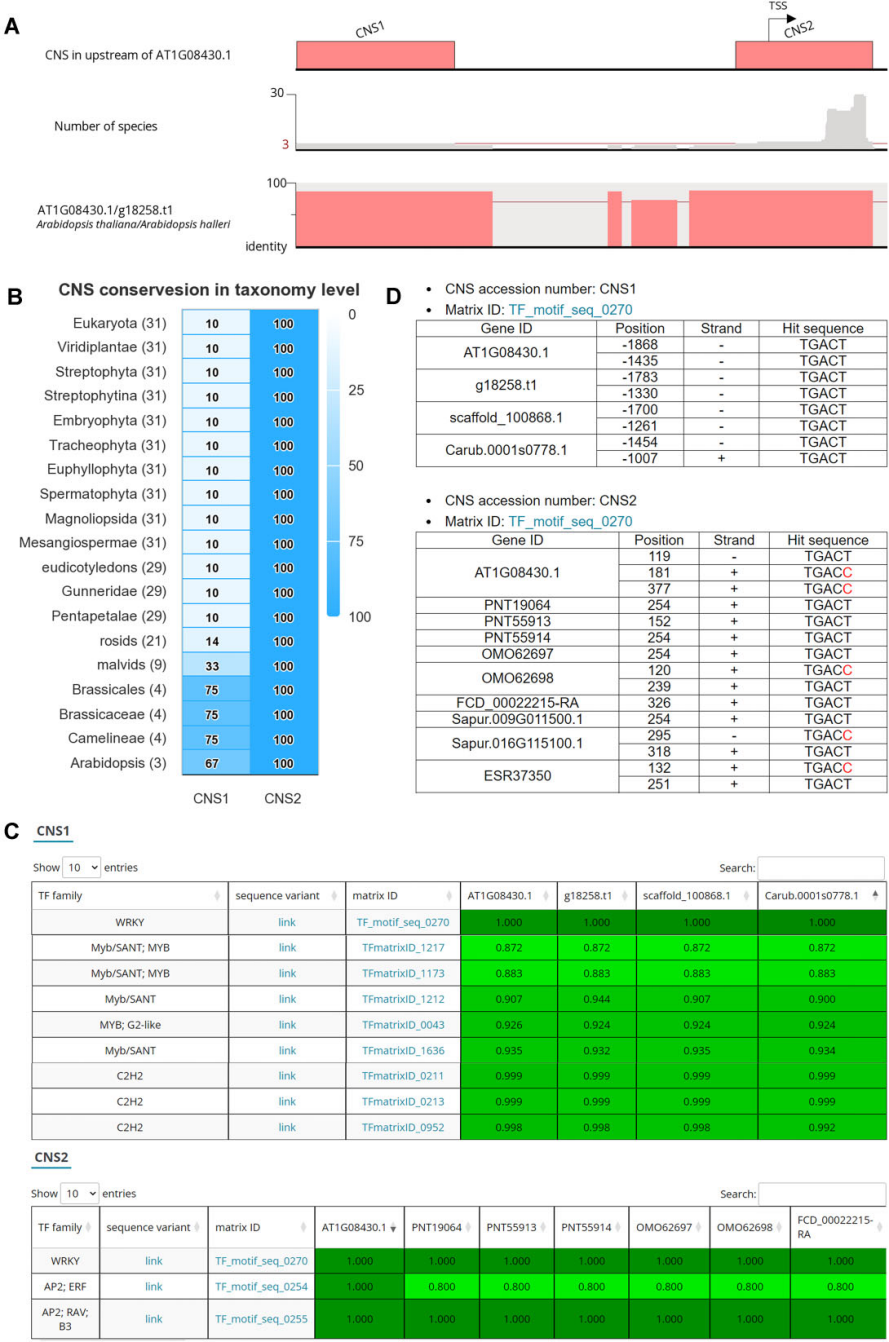
Results of CNS analysis. (**A**) CNSs located in upstream of *ALMT1* (AT1G08430.1). The lanes, from top to bottom, indicate the positions of CNSs, number of species sharing an upstream sequence and distribution of identity between *ALMT1* and its homologous gene in *A. helleri* (g18258.t1). (**B**) Evolutionary conservation of CNSs within each plant lineage. The level on the heat map indicates the percentage of homologous genes within the lineage that share a particular CNS. (**C**) Partial results pertaining to the prediction of TFBSs within CNS1 (top) and CNS2 (bottom). (**D**) Nucleotide variants of the WRKY-binding matrix TF_motif_seq_0270 within CNS1 (top) and CNS2 (bottom). The nucleotides that differ from the majority are marked in red. *ALMT1*, aluminum-activated malate transporter 1 gene; CNS, conserved non-coding sequence.

Two CNSs (CNS1 and CNS2; Figure 1A) were identified in the upstream regions of *ALMT1* (AT1G08430.1) through a CNS analysis performed using the following parameters: sequence identity ≥70%, *e*-value <0.001, minimum CNS length = 30 nucleotides and minimum number of homologous species = 3. CNS1 (located 1995–1328 bp upstream from the transcriptional start site [TSS] of *ALMT1*) was found to be highly conserved in *Brassicales*. By contrast, CNS2 was identified in all plant lineages with a significant conservated region at the downstream of the TSS (Figure 1A, B). The results of TFBS prediction indicated that the well-known TF WRKY46 can regulate the expression of *ALMT1* by binding to two sites within CNS1 and CNS2, which are consistent with its binding matrix, TF_motif_seq_0270 (Figure 1C) (40). In the analysis of TFBS variants, four plant species were found to exhibit the same WRKY-binding sequence within CNS1; however, a one-nucleotide variant of the WRKY-binding site was observed within CNS2 (Figure 1D). Notably, a key activator of *ALMT1* expression, SENSITIVE TO PROTON TOXICITY1, was not observed within any CNSs; rather, it was found in the promoter region of ALMT1 outside of the two CNSs (Supplementary Figure S1) (39). This finding indicate that the binding sites of this activator may change during evolutionary processes or switch to ensure the use of different binding matrices of the C2H2 zinc finger family (Figure 1C). Additionally, several stress-related TF families, such as AP2/ethylene responsive factor and MYB protein, shared the binding sites within the CNS1 and CNS2 (41,42). These TFs hold promise as candidate regulators of *ALMT1* expression. In summary, this case study suggests that CNS analysis can facilitate the identification of CNSs and exploration of the combinatorial and nucleotide variants of TFBSs.

### Statistical review and graphical analysis components of the Gene Search function of PCBase 2.0

To increase the intelligibility of ChIP-seq data, the ChIP-seq Statistics function—which helps summarize the numbers of RFs, treatment tags and tissue types in the retrieval results—was incorporated into PCBase 2.0. In addition, two graphical analysis functions—Heat Map Visualization of RF Binding Sites and Peak Visualization—were incorporated into the tool to facilitate the comparison of diverse RFs and experimental conditions (treatment tags and tissue types). Herein, we present a case study of *AP1* to clarify the application of the Gene Search function.

**Figure 2.**
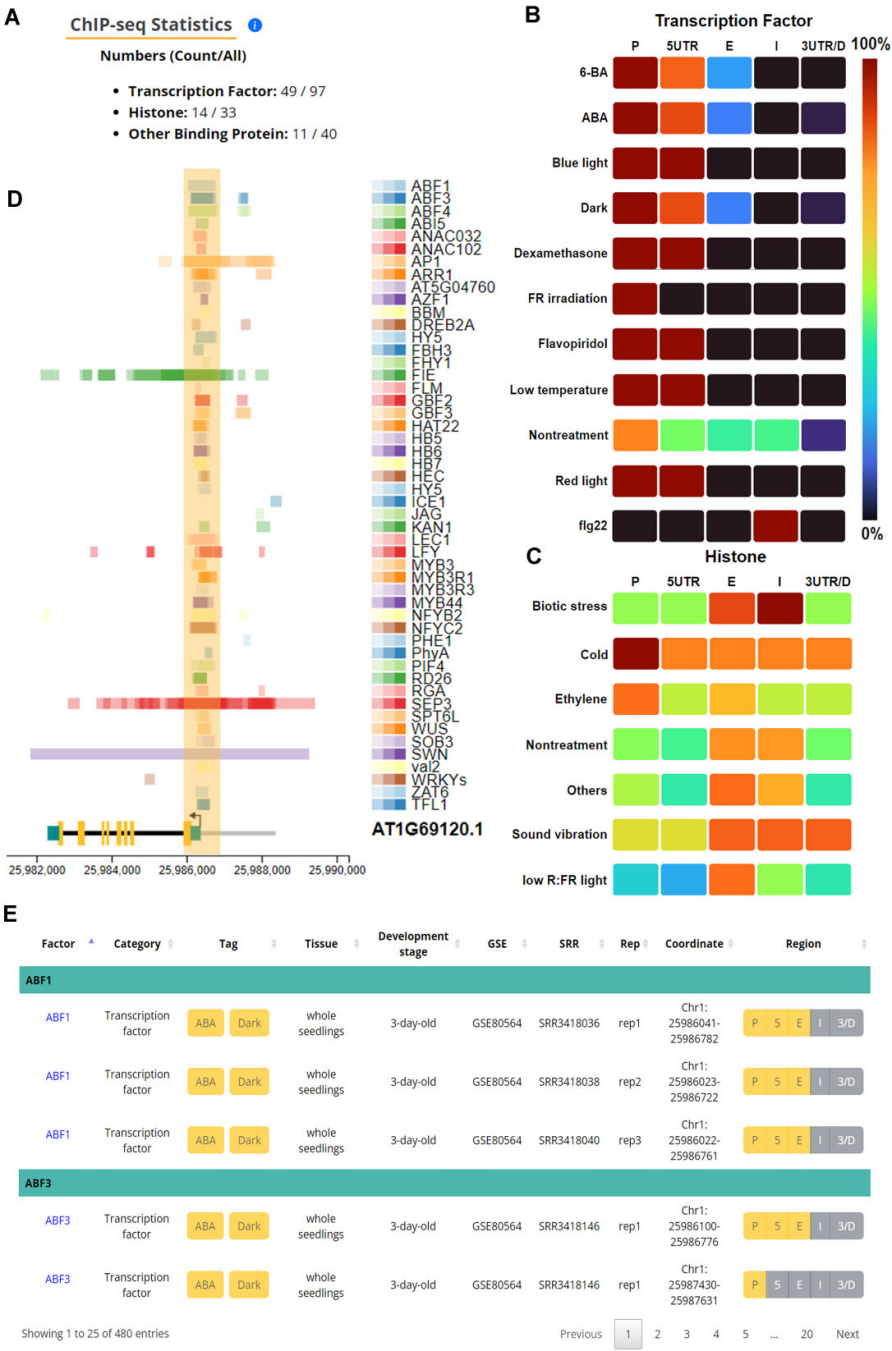
Results of Gene Search function in PCBase 2.0. (**A**) ChIP-seq Statistics, indicating the number of AP1-bound RFs and the total number of RFs in PCBase 2.0 (separated using slashes). (**B**, **C**) Results of the Heat Map Visualization of RF Binding Sites. The binding patterns of histone (B) and TFs (C) were identified on based of five *AP1* regions (P: promoter; 5UTR: 5′ untranslated region; E: exon; I: intron; 3UTR: 3′ untranslated region and D: downstream region). (**D**) Results of Peak Visualization, indicating the binding regions of TFs and a cofactor (TERMINAL FLOWER 1, TFL1) on AP1. The TF-enriched region (hotspot) is marked by the yellow column. (**E**) Partial results of the Table Browser function for ABA-related TFs in ABA-treated 3-day-old (early developmental stage) seedling. ABA, abscisic acid; RF, regulatory factor; TF, transcription factor; ABF1, ABSCISIC ACID RESPONSIVE ELEMENT-BINDING FACTOR 1; ABF3, ABSCISIC ACID RESPONSIVE ELEMENTS-BINDING FACTOR 3; ABF4, ABRE BINDING FACTOR 4; ANAC032, NAC DOMAIN CONTAINING PROTEIN 32; ABI5, ABA INSENSITIVE 5.

*AP1* encodes a MADS-box TF in *A. thaliana* and plays a crucial role in the early stage of floral development (43). *AP1* interacts with the TF LEAFY (LFY) and is essential for meristem transition from the inflorescence meristem to floral meristem (43–45). The expression of *AP1* is activated by LFY and suppressed by the negative regulator TERMINAL FLOWER 1 (45,46). According to the Gene Information page of PCBase 2.0, AP1 is bound by 49 TFs, 14 histones (or histone modifications) and 11 other DNA-binding proteins (Figure 2A). The Heat Map Visualization of RF Binding Sites section illustrates the preferred binding regions of RFs under various treatments or in different tissues. This case study indicated that the TFs primarily bind to promoter regions, whereas histones or histone modifications are enriched in exon and intron regions (Figure 2B, C). The observed difference between TFs and histones in the usage of binding regions is consistent with the finding of a relevant study (9). In further exploration of the RF binding patterns, the result of Peak Visualization revealed a TF hotspot proximal to the TSS of *AP1*; this hotspot with significant TF enrichment is regarded as the core promoter region (Figure 2D) (47). Several flowering-related TFs and cofactors—such as LFY, TERMINAL FLOWER 1, AP1, FLM, JAGGED, SEPALLATA3 (SEP3), KANADI and WUSCHEL—can occupy the *AP1* promoter (45,46,48–52). Of the TFs that target AP1, SEP3 and two core polycomb repressive complex 2 components—FERTILIZATION-INDEPENDENT ENDOSPERM (FIE) and SWINGER (SWN)—exhibit similar occupancy patterns, which are distinct from those of other TFs on *AP1* (9,53). The binding regions of SEP3, FIE and SWN were found in not only the promoter region but also the downstream of the TSS. Furthermore, the combination of the Peak Visualization and Table Browser functions unveiled novel potential regulatory relationships between AP1 and abscisic acid (ABA)–related TFs in ABA-treated 3-day-old seedlings (Figure 2D, E). The results of Peak Visualization revealed that several AP1-bound ABA-related TFs, such as ABSCISIC ACID RESPONSIVE ELEMENT-BINDING FACTOR 1, ABSCISIC ACID RESPONSIVE ELEMENTS-BINDING FACTOR 3, ABRE BINDING FACTOR 4, NAC DOMAIN CONTAINING PROTEIN 32 and ABA INSENSITIVE 5 (54–57). This finding indicates that ABA-related TFs can regulate the expression of *AP1* in the early stage of development.

Overall, this case study suggests that the incorporation of statistical review and graphical analysis components into the Gene Search function can help users compare the genome occupancy patterns of RFs under various treatment clusters and for different tissue types. Furthermore, the integration of publicly available ChIP-seq data and facilitation of the visualization of comprehensive regulatory relationships can help users effectively explore the pivotal regulatory regions on the gene of interest.

### Functional enrichment analysis of the Protein Search function of PCBase 2.0

In addition to the gene-centered (Gene Search) function, the Protein Search function of PCBase 2.0 was optimized on the basis of the statistical data and functional enrichment analysis results. The updated PCBase 2.0 offers the ChIP-seq Statistics function, which facilitates a rapid overview of protein data. Through functional enrichment analysis, users can explore the dynamic transcriptional relationships between a protein of interest and its target genes under various conditions.

**Figure 3.**
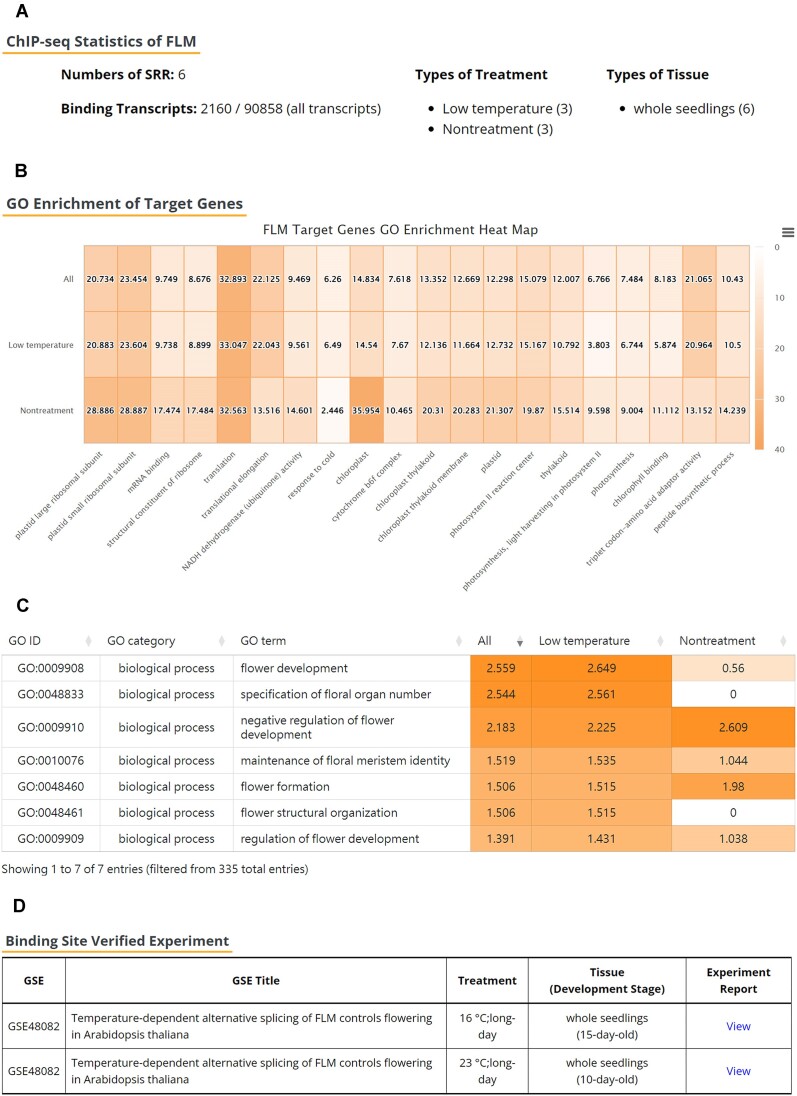
Results of Protein Search function in PCBase 2.0. (**A**) ChIP-seq Statistics of FLM. ‘Number of SRR’ represents FLM-associated ChIP-seq samples. ‘Binding transcript’ refers to the number of transcripts bound by FLM. The numbers of samples in each treatment/tissue cluster are presented within parentheses. (**B**) Results of GO Enrichment of Target Genes. The values presented in the heat map indicate the significance of each GO term, as calculated using − log(*P* value). (**C**) Flowering-related GO terms from the full enrichment analysis result. The flowering-related terms were obtained from the searchable table by typing the keywords ‘flower’ and ‘floral’. The values presented in the table indicate the enrichment levels, which were calculated using −log(*P* value). (**D**) Results of the Binding Site Verified Experiment. FLM, FLOWERING LOCUS M.

Herein, we present a case study of *FLM* derived from *A. thaliana*. *FLM* encodes a MADS-box TF, which inhibits flowering and plays a vital role in the temperature-responsive mechanism underlying flowering. A study reported that *FLM* produces two main splicing variants, FLM-β and FLM-δ, which respond to the regulation of flowering time (58). A low-temperature environment can favor the formation of FLM-β and thus promote the delayed-flowering phenotype (48,58,59). The aforementioned study also indicated the pleiotropic functions of FLM during the vegetative stage; these functions include the regulation of leaf temperature, photosynthetic rate and leaf mass (60). Use of the Protein Search function of the updated database reveal that 2160 transcripts were bound by FLM under low-temperature and nontreatment conditions (Figure 3A). Compared with the results of the GO Enrichment of Target Genes under the aforementioned conditions, the target genes of FML under low-temperature condition exhibited a higher level of significant enrichment in the ‘response to cold’ GO term (Figure 3B). In addition to the cold response-related GO term, other top 20 GO terms also showed the enrichment differences between two different treatments such as ‘mRNA binding’, ‘structural constituent of ribosome’, ‘translational elongation’ and ‘NADH dehydrogenase (ubiquinone) activity’. These enriched terms indicated that the low-temperature conditions may affect the FLM protein in regulating the transcription and translation activities. Notably, 9 of the top 20 GO terms were related to chloroplast or photosynthesis function in 10- and 15-day-old (developmental stage) samples; this observation is consistent with the ability of FLM to modulate leaf physiology during vegetative growth (Figure 3B and D) (60). As mentioned above, FLM could cause a delayed flowering phenotype under low-temperature conditions. A comprehensive GO enrichment analysis revealed that the flowering-related GO terms noted under a low-temperature condition were more significant than those noted under a nontreatment condition; thus, the target genes were more strongly related to flowering regulation under the low-temperature condition than under the nontreatment condition (Figure 3C). The optimized Protein Information function can facilitate the analysis of dynamic regulations, thus enabling users to investigate potential regulatory functions under various conditions.

## Conclusion

Unlike resources similar to PlantPAN 4.0, the updated PlantPAN 4.0 database offers comprehensive functions for the analysis of plant promoters and the reconstruction of gene regulatory networks (Supplementary Table S2). To offer key insights into the regulatory features present within plant genomes, CNS identification and TFBS prediction tools have been combined into the CNS analysis function of PlantPAN 4.0. The improved version of PCBase 2.0 can enhance users’ ability to understand the gene regulation data derived from numerous ChIP-seq data sets, thus facilitating the comparison of genome occupancy across RFs and experimental conditions. PlantPAN 4.0 may help researchers explore the evolutionary conservation of gene regulation in plant genomes and construct frameworks for the dynamic regulation of RFs and the coordination between TFs and histones.

## Supplementary Material

gkad945_supplemental_files

## Data Availability

The PlantPAN 4.0 is available via a web interface and is free to all interested users, at http://PlantPAN.itps.ncku.edu.tw/.
