# Supplementary material for: PlantPAN 4.0: updated database for identifying conserved non-coding sequences and exploring dynamic transcriptional regulation in plant promoters
Source: Nucleic Acids Res. 2023 Oct 28;52(D1):D1569–78. doi: 10.1093/nar/gkad945 (PMC10767843; doi:10.1093/nar/gkad945)
Supplement: gkad945_supplemental_files [file gkad945_supplemental_files.zip › Supplementary Data.pdf]

[SUPPLEMENTARY DATA]

ChIP-seq Data Processing

ChIP-seq analysis was performed as previously described with the following adjustments (1). Raw samples were downloaded and converted to FASTQ format by using SRA toolkit (version 2.8.2-1, https://www.ncbi.nlm.nih.gov/sra). The adapters of reads were trimmed by using cutadapt (version 1.16) (2) and the low quality reads were filtered out by using FASTX-Toolkit (version 0.0.13, http://hannonlab.cshl.edu/fastx_toolkit/). The trimmed reads were mapped to the updated genome version in PlantPAN 4.0 using Bowtie 2 (version 2.4.5) with the default parameters. Before the peak calling step, the resultant SAM-formatted files were sorted and filtered out the reads by using SAMtools (version 1.4). The single-end reads were sorted and the reads with low-quality labels were removed by 'samtools sort' and 'samtools views –F 1284'. As for paired-end samples, 'samtools –F 1804' and additional 'samtools fixmate -r' were used for the data processing. Picard (version 2.18.5, http://broadinstitute.github.io/picard/) was used to discard the duplicate reads. The reads data with a low mapping rate (< 60%) were discarded. For the peak calling step, the MACS2 (version 2.1.0) program was used to identify the regulatory factors binding sites. The computational pipeline was based on the ChIP-seq guidelines developed by ENCODE consortium. Transcription factors, other DNA-binding proteins, and the histone modifications (i.e., 'H2AFZ', 'H3ac', 'H3K27ac', 'H3K4me2', 'H3K4me3', and 'H3K9ac') were perceived as narrow-peak factors. The rest histone and histone modifications were perceived as broad-peak histone. *De novo* motif discovery was conducted by MEME-ChIP in MEME-SUITE (version 4.12.0)(3). The top three most potential position weight matrices from MEME and the advanced algorithm STREME were used for the

promoter analysis function (4).

References

1. Chow, C.-N., Lee, T.-Y., Hung, Y.-C., Li, G.-Z., Tseng, K.-C., Liu, Y.-H., Kuo, P.-L., Zheng, H.-Q. and Chang, W.-C. (2019) PlantPAN3.0: a new and updated resource for reconstructing transcriptional regulatory networks from ChIP-seq experiments in plants. *Nucleic Acids Research*, **47**, D1155-D1163.
2. Martin, M. (2011) Cutadapt removes adapter sequences from high-throughput sequencing reads. *EMBnet.journal; Vol 17, No 1: Next Generation Sequencing Data Analysis*.
3. Machanick, P. and Bailey, T.L. (2011) MEME-ChIP: motif analysis of large DNA datasets. *Bioinformatics*, **27**, 1696-1697.
4. Bailey, T.L. (2021) STREME: accurate and versatile sequence motif discovery. *Bioinformatics*, **37**, 2834-2840.
