# Supplementary material for: PlantPAN 4.0: updated database for identifying conserved non-coding sequences and exploring dynamic transcriptional regulation in plant promoters
Source: Nucleic Acids Res. 2023 Oct 28;52(D1):D1569–78. doi: 10.1093/nar/gkad945 (PMC10767843; doi:10.1093/nar/gkad945)
Supplement: gkad945_supplemental_files [file gkad945_supplemental_files.zip › Supplementary Figure S1.pdf]

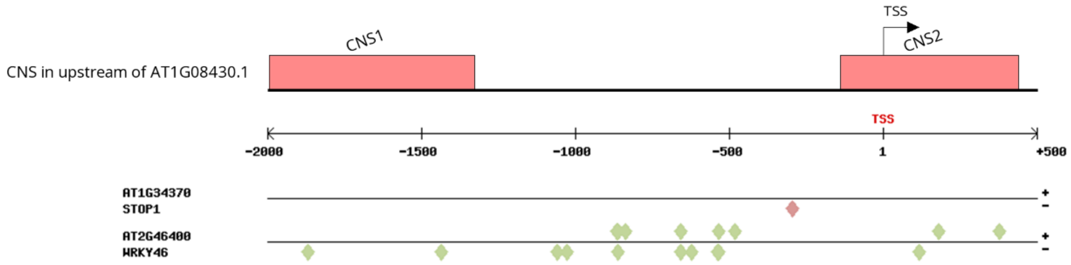

Supplementary Figure S1. Two CNSs and TFBSs of STOP1 and WRKY46 in the upstream regions of ALMT1 gene (AT1G08430.1). The TFBSs of STOP1 and WRKY46 were predicted by using promoter analysis function in 'Gene Search' of PlantPAN 4.0.
