# Supplementary material for: PlantPAN 4.0: updated database for identifying conserved non-coding sequences and exploring dynamic transcriptional regulation in plant promoters
Source: Nucleic Acids Res. 2023 Oct 28;52(D1):D1569–78. doi: 10.1093/nar/gkad945 (PMC10767843; doi:10.1093/nar/gkad945)
Supplement: gkad945_supplemental_files [file gkad945_supplemental_files.zip › Supplementary Table S2.pdf]

Supplementary Table S2. A statistical and functional comparison between PlantPAN and similar resources.

	**PlantPAN 4.0**	**PlantPAN 3.0**[a]	**dbCNS**[b]	**PlantRegMap**[c]	**ChIP-hub**[d]	**ChIPBase v3.0**[e]	**ReMap 2022**[f]
**No. of species in this database**	**115**	7	0 (180)[g]	63	48	1 (5)[g]	1 (4)[g]
**Functions for identification of CNSs**							
**Custom parameters for identification of CNSs**	**Yes**	No	Yes	Yes	NA	NA	NA
**Graphical results (phylogenetic tree of homologous genes, protein domain structures, and genomic similarities of CNSs across multiple promoters)**	**Yes**	No	No	No	NA	NA	NA
**TFBS prediction in CNSs**	**Yes**	Yes	No	No	NA	NA	NA
**Combinational and nucleotide variants of TFBSs in CNSs**	**Yes**	No	No	No	NA	NA	NA
**Functions for ChIP-seq collection**							
**Experimental condition clusters**	**Yes (tissue/condition)**	No	NA	Yes (organ/condition)	No	Yes (tissue/cell)	Yes (tissue/cell)
***De novo* motif discovery**	**Yes**	Yes	NA	No	No	Yes	No
**TFBS prediction by using ChIP-seq derived matrices**	**Yes**	Yes	NA	No	No	Yes	No
**Overview of regulation on target genes (binding preferences for regulatory factors/conditions and peak visualization for all ChIP-seq data)**	**Yes**	No	NA	No	No	No	No
**Functional enrichment analysis of TFs based on ChIP-seq target genes**	**Yes**	No	NA	No	No	Yes	No
**Genomic landscapes of open chromatin regions**	**Yes**	No	NA	Yes	Yes	No	No

[a]: PlantPAN 3.0: http://PlantPAN.itps.ncku.edu.tw/ (Chow et al., *Nucleic Acids Res.*, 2019)
[b]: dbCNS: http://yamasati.nig.ac.jp/dbcns (Inoue et al., *Mol Biol Evol.*, 2021)
[c]: PlantRegMap: http://plantregmap.cbi.pku.edu.cn/ (Tian et al., *Nucleic Acids Res.*, 2019)
[d]: ChIP-hub: https://biobigdata.nju.edu.cn/ChIPHub/ (Fu et al., *Nat Commun.*, 2022)
[e]: ChIPBase v3.0: https://rnasysu.com/chipbase3/ (Huang et al., *Nucleic Acids Res.*, 2022)
[f]: ReMap 2022: https://remap.univ-amu.fr (Hammal et al., *Nucleic Acids Res.*, 2021)
[g]: The number of data for plant species is shown without brackets, whereas the total number for both plant and non-plant species is indicated within brackets.
